# Tuina-Focused Integrative Chinese Medical Therapies for Inpatients with Low Back Pain: A Systematic Review and Meta-Analysis

**DOI:** 10.1155/2012/578305

**Published:** 2012-12-26

**Authors:** Ling Jun Kong, Min Fang, Hong Sheng Zhan, Wei An Yuan, Jiang Hui Pu, Ying Wu Cheng, Bo Chen

**Affiliations:** ^1^Yueyang Hospital of Integrated Traditional Chinese and Western Medicine, Shanghai University of Traditional Chinese Medicine, Shanghai 200437, China; ^2^Research Institute of Tuina, Shanghai Academy of Traditional Chinese Medicine, Shanghai 201203, China; ^3^Department of Orthopedics, Shuguang Hospital, Shanghai University of Traditional Chinese Medicine, Shanghai 201203, China

## Abstract

*Objective*. To evaluate the effectiveness of Tuina-focused integrative Chinese medical therapies (TICMT) on inpatients with low back pain (LBP). *Methods*. 6 English and Chinese databases were searched for randomized controlled trials (RCTs) of TICMT for in-patients with LBP. The methodological quality of the included RCTs was assessed based on PEDro scale. And the meta-analyses of TICMT for LBP on pain and functional status were conducted. *Results*. 20 RCTs were included. The methodological quality of the included RCTs was poor. The meta-analyses' results showed that TICMT had statistically significant effects on pain and functional status, especially Tuina plus Chinese herbal medicine (standardised mean difference, SMD: 1.17; 95% CI 0.75 to 1.60 on pain; SMD: 1.31; 95% CI 0.49 to 2.14 on functional status) and Tuina plus acupuncture (SMD: 0.94; 95% CI 0.38 to 1.50 on pain; SMD: 0.53; 95% CI 0.21 to 0.85 on functional status). But Tuina plus moxibustion or hot pack did not show significant improvements on pain. And the long-term evidence of TICMT was far from sufficient. *Conclusions*. The preliminary evidence from current studies suggests that TICMT might be effective complementary and alternative treatments for in-patients with LBP. However, the poor methodological quality of the included RCTs means that high-quality RCTs with long follow-up are warranted.

## 1. Introduction

Low back pain (LBP) and related disability are one of the major public health problems worldwide, which represent a great financial burden in the form of direct costs resulting from the loss of work and medical expenses, as well as indirect costs [1]. And the prevalence of LBP is quite high and increases according to the time span considered. The point prevalence of bothersome LBP has been estimated at 25%, whereas the 1-year prevalence has been estimated at 50% and the lifetime prevalence has been estimated at 85% [2–4]. Therefore, the adequate treatment of LBP is an important issue for patients, treating clinicians, and healthcare policy makers. 

Tuina, a manual therapy in traditional Chinese medicine, emphasizes anatomy and physiology when used for neuromusculoskeletal disorders. Currently it is widely used for the treatment of LBP. Tuina procedures for LBP are combined soft-tissue manipulation with spinal manipulation. Soft-tissue manipulation is similar to massage, including stroking, kneading, and percussion. Spinal manipulation, on the other hand, is quite similar to mobilization and other adjustment techniques. These techniques can involve a manual procedure without thrust, during which a joint normally remains within its physiological range of motion. Alternatively, they can involve a manual procedure directed thrust to move a joint past the physiological range of motion, without exceeding the anatomical limit [5]. The clinical practice guidelines have formed moderate recommendations of massage, mobilization, and manipulation for LBP [6, 7]. Some systemic reviews also concluded that these manual therapies might be beneficial for LBP [8–11]. But the evidence is only for single application of these manual therapies.

In the last decade, a mass of hospitals have adopted Tuina-focused integrative Chinese medical therapies (TICMT) in the management of LBP for better effectiveness in China, which consist of Tuina combined with other traditional Chinese medical therapies including Chinese herbal medicine, acupuncture, moxibustion, and hot pack. In addition, a number of clinical studies on TICMT have been rolled out and published [12]. However, the evidence from systematic reviews on TICMT for LBP is marginal. Therefore, we performed a systematic review of all currently available data and conducted quantitative meta-analyses of TICMT for in-patients with LBP to determine whether TICMT are effective complementary and alternative treatments for in-patients with LBP.

## 2. Methods

### 2.1. Search

The following electronic databases were searched from January 2001 to June 2012: PubMed, EMBASE, Cochrane Library, China Knowledge Resource Integrated Database (CNKI), Weipu Database for Chinese Technical Periodicals (VIP), and Wanfang Data. The first search terms were low back pain, lumbago, lumbar disc herniation, lumbar sprain, backache, back pain, or dorsalgia. The second terms were Tuina, massage, mobilization, or spinal manipulation. The third search terms were acupuncture, electroacupuncture, herbal medicine, moxibustion, or hot pack, and the last search term was random. We combined these four terms for text word searches of titles and abstracts. No restrictions on publication status were imposed. The complete search strategies for each database were shown in Appendix A. 

### 2.2. Study Selection

Randomized controlled trials (RCTs) of TICMT for in-patients with LBP were included. There were no limitations on the participant's age, gender, or nationality. The included integrative therapies were Tuina combined with other traditional Chinese medical therapies including Chinese herbal medicine (herbal decoctions and herbal injections), acupuncture (manual acupuncture and electroacupuncture), moxibustion, and hot pack. Control treatments included any independent traditional Chinese medical therapy, placebo, waiting list controls, and integrative treatments without any manual therapy. The main outcomes of interest were pain and functional status. 

Trials were excluded if any of the following were identified: (1) if the participants were outpatients; (2) if controlled treatment was an integrative therapy including any manual therapy. In this case, it would be impossible to evaluate the specific effect of Tuina combined with other traditional Chinese medical therapies; and (3) if the information about the outcome measures was not clearly reported. 

### 2.3. Data Abstraction

Two authors extracted data independently according to predefined criteria including the first author, year of the study, the mean duration of LBP, sample size, the mean age of participants, the duration of treatments, the follow-up time, main outcome assessments, interventions of TICMT and control group, and the main conclusion (mean improvements). Any discrepancies were discussed until the authors reached consensus.

### 2.4. Methodological Quality Assessment

The methodological quality of RCTs was assessed independently by two authors by PEDro scale, which is based on the Delphi list and has been reported to have a fair to good reliability for RCTs of the physiotherapy in systematic reviews. This scale consists of 11 criteria being (1) study eligibility criteria specified, (2) random allocation of subjects, (3) concealed allocation, (4) measure of similarity between groups at baseline, (5) subject blinding, (6) therapist blinding, (7) assessor blinding, (8) less than 15% dropouts, (9) intention-to-treat analysis, (10) between-group statistical comparisons, and (11) point measures and variability data. Criteria (2)–(11) were used to calculate the PEDro score. Each criterion was scored as either 1 or 0 according to whether the criteria was met or not, respectively. The scores are summed and a higher score represents a better methodological quality. A cut point of 6 on the PEDro scale was used to indicate high quality studies as this has been reported to be sufficient to determine high quality versus low quality in previous studies [33]. If additional data or clarification was necessary, we contacted the study authors. And disagreements were resolved by discussions among the authors.

### 2.5. Data Synthesis and Analysis

The mean change in outcome measures between the end of the final intervention and the baseline was used to assess the difference between TICMT group and control group in the meta-analyses. Standardised mean differences (SMDs) were used because the studies measured the outcomes based on different scales (e.g., VAS 0–10 and VAS 0–100). And SMDs and 95% confidence intervals (CIs) were calculated in the meta-analysis. In studies that involved more than one control group, the authors restricted our analyses to TICMT and each control group. Summary estimates of the treatment effect were calculated using the random effects model to account for the expected heterogeneity. Cochrane's *Q* test and *I* ^2^ were used to assess statistical heterogeneity. The authors determined that there was considerable heterogeneity when Cochrane's *Q* test result was determined with *P* < 0.10, and *I* ^2^ was above 75%. The Cochrane Collaboration software (Review Manager Version 5.0 for Windows; Copenhagen: The Nordic Cochrane Centre) was used for the meta-analyses. And the results of study characteristics are presented as mean ± standard deviation (SD) or the range of variation. 

## 3. Results

### 3.1. Study Selection

**Figure 1:**
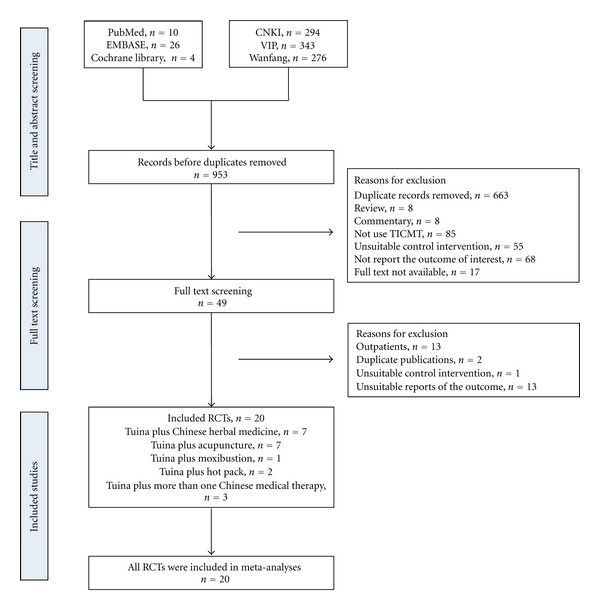
Study selection process. RCTs: randomized controlled trials and TICMT: Tuina-focused integrative Chinese medical therapies.

We identified 953 records from English and Chinese databases. After the initial titles and abstracts screening, we excluded 904 because of a large number of duplicate records from three Chinese databases (CNKI, VIP, and Wanfang) and some reports did not met the inclusion criteria. We retrieved and reviewed 49 full articles. 20 RCTs were eligible [13–32]. In excluded studies, the trials were excluded due to outpatients (*n* = 13), duplicate publications (*n* = 2), unsuitable control intervention (*n* = 1), and unsuitable reports of the outcome (*n* = 13). And all RCTs were included in meta-analyses. The study selection process was summarized in Figure 1.

### 3.2. Study Characteristics

Twenty eligible studies included 2147 subjects with the mean age of 43. And all studies were conducted in China between 2005 and 2012. The disease duration ranged from 1 day to 10 years, and the study duration ranged from 3 days to 8 weeks. The time and session of Tuina treatment were 26.3 ± 4.4 minutes (ranging from 20 to 30 minutes) and 17.5 ± 10.5 (ranging from 3 to 42), respectively. In combined traditional Chinese therapies, the number of Chinese herbal medicine every day ranged from 1 to 3, and acupuncture points were 6.2 ± 3.1 (ranging from 1 to 10). The hot pack time ranged from 30 to 360 minutes. The follow-up time ranged from 4 to 24 weeks.

Of twenty RCTs, 7 RCTs assessed the effectiveness of Tuina plus Chinese herbal medicine for in-patients with LBP [13–19], 7 RCTs assessed the effect of Tuina plus acupuncture [20–26], 2 RCTs assessed the effect of Tuina plus hot pack [27, 28], and one assessed the efficacy of Tuina plus moxibustion [29]. The others assessed the effectiveness of Tuina plus more than one Chinese medical therapy [30–32]. The control therapies contained Tuina, Chinese herbal medicine, acupuncture, moxibustion, traction, electromagnetic therapy, or integrated treatments (including Chinese herbal medicine plus traction, Chinese herbal hot pack plus traction, Chinese herbal hot pack plus electromagnetotherapy, and traction plus infrared radiation). In outcome assessments, visual analog scale (VAS) was used for pain, and the Oswestry disability index (ODI) or Japanese orthopaedic association score for low back pain (JOA) was used for functional status. The characteristics of all studies were summarized in Table 1.

### 3.3. Methodological Quality

The quality scores were presented in Table 2. The quality scores ranged from 5 to 8 points out of a theoretical maximum of 10 points. Although the predetermined cutoff 6 was exceeded by most studies included, it did not indicate that they were considered to be of high quality, because most of them (80% of studies) were at the limit of the cutoff with scores of 6. And there were serious flaws in concealed allocation (90% of studies), subjects blinded (100% of studies), therapists blinded (100% of studies), and assessors blinded (95% of studies). In addition, two studies were failed in random allocation, because the patients were randomly allocated by hospital record number. In other items on PEDro scale, the studies showed higher methodological quality involving measure of similarity between groups at baseline, less than 15% dropouts, intention-to-treat analysis, between-group statistical comparisons, and point measures and variability data. 

### 3.4. Quantitative Data Synthesis

#### 3.4.1. Effects of TICMT on Pain

Six RCTs tested the effectiveness of Tuina plus Chinese herbal medicine on pain for LBP compared with Tuina [13–15, 19], Tuina (or Chinese herbal medicine) [17], and Chinese herbal medicine plus traction [16]. And the meta-analysis showed favorable effects of Tuina plus Chinese herbal medicine on pain (*n* = 765; SMD: 1.17; 95% CI 0.75 to 1.60; *P* < 0.00001; heterogeneity: *χ* ^2^ = 42.05, *P* < 0.00001, *I* ^2^ = 86%; Table 3).

Five trials assessed the effect of Tuina plus acupuncture on pain for LBP versus Tuina [20], Tuina (or acupuncture) [22], electroacupuncture [23], traction [24], and electromagnetotherapy plus Chinese herb hot pack [25]. The meta-analysis showed superior effects of Tuina plus acupuncture on pain relief (*n* = 640; SMD: 0.94; 95% CI 0.38 to 1.50; *P* = 0.001; heterogeneity: *χ* ^2^ = 55.70, *P* < 0.00001, *I* ^2^ = 91%; Table 3). And one study tested the effectiveness of Tuina plus acupuncture and Chinese herbal medicine on pain for LBP compared with Tuina [31]. The meta-analysis also showed significant effects (*n* = 60; SMD: 1.61; 95% CI 1.03 to 2.20; *P* < 0.00001; Table 3).

One RCT tested the effectiveness of Tuina plus moxibustion on pain for LBP versus Tuina or moxibustion [29]. The meta-analysis did not shown favorable effects of Tuina plus moxibustion on pain reduction (*n* = 120; SMD: 0.42; 95% CI −0.17 to 1.02; *P* = 0.16; heterogeneity: *χ* ^2^ = 2.67, *P* = 0.10, *I* ^2^ = 63%; Table 3). In addition, Tuina plus hot pack did not show better effects on pain than Tuina (*n* = 120; SMD: −0.77; 95% CI −1.14 to −0.39; *P* < 0.0001; Table 3) [28].

#### 3.4.2. Effects of TICMT on Functional Status

Two RCTs tested the effect of Tuina plus Chinese herbal medicine on functional status for LBP compared with Tuina [13, 18]. And the meta-analysis showed favorable effects of Tuina plus Chinese herbal medicine on functional status (*n* = 223; SMD: 1.31; 95% CI 0.49 to 2.14; *P* = 0.002; heterogeneity: *χ* ^2^ = 5.89, *P* = 0.02, *I* ^2^ = 83%; Table 4).

Two trials assessed the effect of Tuina plus acupuncture on functional status versus traction plus infrared radiation [21] or acupuncture [26]. Two studies maintained that in-patients in Tuina plus acupuncture group experienced more obvious improvements on functional status. And the meta-analysis also showed superior effects of Tuina plus acupuncture on functional status (*n* = 160; SMD: 0.53; 95% CI 0.21 to 0.85; *P* = 0.001; heterogeneity: *χ* ^2^ = 0.65, *P* = 0.42, *I* ^2^ = 0%; Table 4). In addition, Tuina plus Chinese herbal hot pack showed better effects on functional status than Tuina (*n* = 40; SMD, 2.82; 95% CI 1.92 to 3.72; *P* < 0.00001; Table 4) [27].

Two trials assessed the effect of Tuina plus more than one Chinese medical therapy on functional status for LBP versus traction plus Chinese herbal hot pack [30] and Tuina [32]. Tuina coupled with traction and Chinese herbal hot pack and Tuina coupled with acupuncture and moxibustion were, respectively, employed in two trials. And the meta-analysis showed favorable effects of Tuina plus more than one Chinese medical therapy on functional status (*n* = 203; SMD, 2.58; 95% CI 1.48 to 3.69; *P* < 0.00001; heterogeneity: *χ* ^2^ = 8.42, *P* = 0.004, *I* ^2^ = 88%; Table 4).

### 3.5. Long-Term Effects of TICMT

Three studies observed the long-term effect of TICMT for in-patients with LBP. But only one trial reported that TICMT group (Tuina plus acupuncture) experienced better improvements on functional status compared with acupuncture (recurrence rate, 6.2% versus 25.9%) [26]. The other two did not show detailed results [13, 22].

## 4. Discussion

In summary, there are encouraging results suggesting that TICMT has short-term effects on improving pain and functional status of in-patients with LBP, especially Tuina plus Chinese herbal medicine or acupuncture. But the quality of the included studies was generally poor. And Tuina plus hot pack or moxibustion did not show better effects on pain relief, which might be explained by the fact that there are relatively fewer eligible studies. In addition, the studies of long-term effects of TICMT were extremely insufficient. Consequently, interpretation of these positive findings should be cautions. 

Our positive results concur with some relevant clinical guidelines and systematic reviews. The clinical guideline from the United States in 2007 found the moderate-quality evidence to support the efficacy of massage and spinal manipulation for the management of LBP [6]. The clinical guidelines from Belgium in 2006 [4] and United Kingdom in 2009 [7] also found the moderate-quality evidence for spinal manipulation and recommended offering a maximum of 9 sessions of spinal manipulation over a period of up to 12 weeks. And the systematic review including 13 RCTs concluded that massage might be beneficial for subacute and chronic nonspecific LBP, and the massage also showed the long-term effect (at least 1 year) [8]. Some systematic reviews [34–36] and clinical guidelines [6, 7] also found the evidence to support the efficacy of acupuncture with respect to improvements on pain and function. In addition, there were some recommendations of herbal medicines for LBP in systematic reviews [37, 38] and clinical guidelines [3, 6]. Although the evidence is only for single application of these therapies for LBP, they partly supported the efficacy of TICMT for the management of LBP. Comparing with these systematic reviews and clinical guidelines, there were some key strong points in our systematic review. We assessed the integrated effect of TICMT for LBP by the qualitative review and quantitative meta-analyses for the first time. Although TICMT were widely used for the in-patients with LBP in Chinese hospitals, the evidence from systematic reviews was marginal. We, on the other hand, separately assessed the effect of Tuina plus Chinese herbal medicine, Tuina plus acupuncture, Tuina plus moxibustion, Tuina plus hot pack, and Tuina plus more than one Chinese medical therapy. And the outcomes of interest contained pain and functional status. So our systematic review provided stronger evidence of TICMT for LBP. 

### 4.1. Limitations of the Review

There are several limitations in our study. First, the distorting effects of publication and location bias on systematic reviews and meta-analyses are well documented [39, 40]. We are confident that our search strategy located all relevant studies. However, some degree of uncertainty remains. Another possible source of bias is that more negative trials of TICMT for in-patients with LBP may be never published in the peer-reviewed literature. Secondly, the quality of the eligible studies was generally poor, which indicated an unclear risk of bias resulting from insufficient reporting of methodological components in the trials. There were serious flaws in concealed allocation and blind methods in the eligible trials, which may have created potential performance biases and detection biases, as patients and assessors might have been aware of the therapeutic interventions. And improper blinding is likely to have led to an overestimation of the effect. In addition, our review also may be affected by the heterogeneity of traditional Chinese therapies. Based on traditional Chinese medicine theory, interventions were designed to be adapted according to the specific presentations of LBP, so these interventions were different in frequency and force of Tuina, type and dose of Chinese herbal medicine, and number of acupuncture points. Last, all eligible RCTs were conducted in China, which may be due largely to the acceptability and service restrictions of traditional Chinese medical therapies in the other countries.

### 4.2. The Possible Rationale of TICMT for LBP

Assuming that TICMT were beneficial for LBP, the complex interplay of both physical and mental modes may provide a possible rationale. The manual therapy delivered to soft and connective tissues may induce local biochemical changes that modulate local blood circulation, improve muscle flexibility, intensify the movement of lymph, and loosen adherent connective tissue, which may alternately improve reuptake of local nociceptive and inflammatory mediators [41, 42]. These effects may subsequently influence neural activity at the spinal cord segmental level, thereby modulating the activities of cerebral cortex that improve pain and dysfunction due to LBP [43]. In addition, manual therapy may impact the primary afferent neurons from paraspinal tissues, the motor control system, and pain processing [44]. Chinese herbal medicine and acupuncture may strengthen the effectiveness of these manual therapies in local blood circulation and nociceptive and inflammatory mediators, because isolated components of the Chinese herbs have anti-inflammatory, antilipidemic, antioxidant, and immune modulation properties [45]. And the acupuncture also works through the central nervous system by stimulating the production of endorphins and neurotransmitters that modulate nociception and other involuntary bodily functions, or through the gate control theory of pain, in which the nociceptive input is inhibited in the central nervous system in the presence of another type of input [46–48]. It also stimulates vascular and immunomodulatory factors involved as mediators of inflammation [49]. 

## 5. Conclusion

Twenty RCTs were analyzed in our systematic review, evaluating TICMT in management of LBP. The findings from the current studies suggest that TICMT might be effective complementary and alternative treatments for in-patients with LBP. However, the poor quality of the included studies and the shortage of long-term effects of TICMT suggest that the positive evidence is underpowered. Consequently, future studies should adhere to high-quality RCTs with long follow-up for demonstrating the effectiveness of TICMT for in-patients with LBP.

## Figures and Tables

**Table 1:** Randomized controlled trials evaluating the effect of Tuina-focused integrative Chinese medical therapies (TICMT) for low back pain (LBP).

First authors, yr	Mean duration of LBP	Sample size, mean age (yr)	Duration weeks	Follow-up weeks	Main outcome assessments	TICMT group (EG) intervention*	Control group (CG) intervention*	Main conclusion (mean improvements)
Tuina plus Chinese herbal medicine								

Liang (2005) [13]	4–36 mo	49 44–65	8	24	Pain VAS (0–10) ODI	Tuina (24 sessions) plus CHM (3/56 sessions)	Tuina (24 sessions)	VAS: EG (3.65) > CG (2.65) ODI: EG (12.40) > CG (7.10)
Huang (2008) [14]	1–13 days	100 42	3	—	Pain VAS (0–10)	Tuina (25 min/21 sessions) plus CHM (1/21 sessions)	Tuina (25 min/21 sessions)	EG (5.78) > CG (4.79)
Su (2008) [15]	NR	120 40	3 days	—	Pain VAS (0–10)	Tuina (30 min/3 sessions) plus CHM (1/3 sessions)	Tuina (30 min/3 sessions)	EG (4.90) > CG (0.55)
Cai (2010) [16]	NR	96 >16	4	—	Pain VAS (0–10)	Tuina (30 min/24 sessions) plus CHM (2/24 sessions)	CHM (2/24 sessions) plus traction (15 min/24 sessions)	EG (8.15) > CG (5.99)
Wen (2010) [17]	4–20 days	120 NR	3	—	Pain VAS (0–100)	Tuina plus CHM (NR)	(1) Tuina (NR) (2) CHM (NR)	EG (21.40) > CG1 (12.20) EG (21.40) > CG2 (11.10)
Zhang (2011) [18]	36 mo	184 58	5	—	ODI	Tuina (28 sessions) plus injection CHM (1/28 sessions)	Tuina (28 sessions)	EG (51.20) > CG (28.20)
You (2012) [19]	34.8 h	240 30	1	—	Pain VAS (0–10)	Tuina (7 sessions) plus CHM (1/7 sessions)	Tuina (7 sessions)	EG (7.86) > CG (6.32)

Tuina plus acupuncture								

Pang (2006) [20]	9.3 mo	120 45	2	—	Pain VAS (0–10)	Tuina (20 min/14 sessions) plus electroacupuncture (4 AP/14 sessions)	Tuina (20 min/14 sessions)	EG (5.51) > CG (4.45)
Chen (2007) [21]	15.9 mo	60 37	2	—	JOA (0–29)	Tuina (25 min/7 sessions) plus acupuncture (3–8 AP/8 sessions)	Traction plus infrared radiation (30 min /14 sessions)	EG (10.83) > CG (8.16)
Liu (2008) [22]	2 days	105 41	1	4	Pain VAS (0–10)	Tuina (7 sessions) plus acupuncture (1 AP/7 sessions)	(1) Tuina (7 sessions) (2) Acupuncture (1 AP/7 sessions)	EG (4.84) > CG1 (4.32) EG (8.74) > CG2 (4.11)
He (2010) [23]	1 day–10 yr	180 38	2	—	Pain VAS (0–10)	Tuina (6 sessions) plus electroacupuncture (10 AP/10 sessions)	Electroacupuncture (10 AP/10sessions)	EG (3.65) > CG (3.17)
Ke (2011) [24]	>7 days	60 20–65	7 days	—	Pain VAS (0–10)	Tuina (7 sessions) plus electroacupuncture (4–6 AP/7 sessions)	Traction (7 sessions)	EG (4.53) > CG (3.14)
Yang (2011) [25]	5 yr	100 41	15 days	—	Pain VAS (0-10)	Tuina (30 min/15 sessions) plus electroacupuncture (3–5 AP/7 sessions)	Electromagnetic therapy (20 min/1 sessions) plus Chinese herbal hot pack (25 min/15 sessions)	EG (4.70) > CG (1.70)
Zeng (2012) [26]	2.9 yr	100 49	2-3	12	JOA (0–29)	Tuina (20 min/14–21 sessions) plus acupuncture (8-9 AP/14–21 sessions)	Acupuncture (8-9 AP/14–21 sessions)	EG (12.70) > CG (10.60)

Tuina plus hot pack								

Rong (2010) [27]	10.2 mo	40 52	20 days	—	JOA (0–29)	Tuina (20 sessions) plus Chinese herbal hot pack (30 min/40 sessions)	Tuina (20 sessions)	EG (11.32) > CG2 (5.23)
Yuan (2010) [28]	>6 mo	120 38	20 days	—	Pain VAS (0–10)	Tuina (20 sessions) plus hot pack (360 min/20 sessions)	Tuina (20 sessions)	EG (3.65) < CG (4.85)

Tuina plus moxibustion								

Liu (2010) [29]	1.2 yr	90 31	4	—	Pain VAS (0–10)	Tuina (30 sessions) plus moxibustion (3 AP/30 sessions)	(1) Tuina (30 sessions) (2) Moxibustion (3 AP/30 sessions)	EG (3.51) > CG1 (3.21) EG (3.51) > CG2 (1.79)

Tuina plus more than one Chinese medical therapy								

Zhou (2009) [30]	3 days–10 yr	100 44	10 days	—	JOA (0–29)	Tuina (10 sessions) plus traction (30–60 min/20 sessions) and Chinese herbal hot pack (30–45 min/20 sessions)	Traction (30–60/20 sessions) plus Chinese herbal hot Pack (30–45 min/20 sessions)	EG (19.82) > CG2 (9.46)
Wang (2011) [31]	1.2 yr	60 45	30 days	—	Pain VAS (0–10)	Tuina (30 sessions) plus acupuncture (7 AP/30 sessions) and CHM (NR)	Tuina (30 sessions)	EG (5.98) > CG (3.40)
Chen (2012) [32]	—	103 —	6	—	JOA (0–29)	Tuina (30 min/42 sessions) plus acupuncture (10 AP/42 sessions) and moxibustion (3/42 sessions)	Tuina (30 min/42 sessions)	EG (11.1) > CG2 (4)

VAS: Visual analog scale; ODI: the oswestry disability index; CHM: Chinese herbal medicine; NR: no reported; JOA: Japanese orthopaedic association score for low back pain; yr: year; mo: month; h: hour; AP: acupuncture point.

*Intervention dose: number of intervention time/number of sessions, number of acupuncture points/number of sessions, or number of Chinese herbal medicine every day/number of sessions.

**Table 2:** PEDro scale of quality for included trials.

Study	Eligibility criteria	Random allocation	Concealed allocation	Similar at baseline	Subjects blinded	Therapists blinded	Assessors blinded	<15% dropouts	Intention-to-treat analysis	Between-group comparisons	Point measures and variability data	Total
Liang [13]	1	1	0	1	0	0	0	1	1	1	1	6
Pang et al. [20]	1	1	0	1	0	0	0	1	1	1	1	6
Chen et al. [21]	1	1	0	1	0	0	0	1	1	1	1	6
Huang [14]	1	1	0	1	0	0	0	1	1	1	1	6
Liu et al. [22]	1	1	0	1	0	0	0	1	1	1	1	6
Su and Lei [15]	1	1	0	1	0	0	0	1	1	1	1	6
Zhou [30]	1	1	0	1	0	0	0	1	1	1	1	6
Cai [16]	1	1	0	1	0	0	0	1	1	1	1	6
He et al. [23]	1	1	1	1	0	0	1	1	1	1	1	8
Liu [29]	1	1	0	1	0	0	0	1	1	1	1	6
Rong [27]	1	1	0	1	0	0	0	1	1	1	1	6
Wen [17]	1	1	0	1	0	0	0	1	1	1	1	6
Yuan et al. [28]	1	1	1	1	0	0	0	1	1	1	1	7
Ke and Li [24]	1	0	0	1	0	0	0	1	1	1	1	5
Wang et al. [31]	1	1	0	1	0	0	0	1	1	1	1	6
Yang et al. [25]	0	1	0	1	0	0	0	1	1	1	1	6
Zhang et al. [18]	1	1	0	1	0	0	0	1	1	1	1	6
Chen [32]	1	1	0	1	0	0	0	1	1	1	1	6
You and Zhou [19]	0	1	0	1	0	0	0	1	1	1	1	6
Zeng [26]	1	0	0	1	0	0	0	1	1	1	1	5

0: does not meet the criteria; 1: meets the criteria.

**Table 3:** Forest plots of the effect of Tuina-focused integrative Chinese medical therapies (TICMT) on pain of in-patients with low back pain. Box in the line for each study: the mid-point of the box represents the mean effect estimate, which area shows the weight given to the study, and the line represents the confidence intervals of the mean effect estimate. The diamond below these studies represents the overall effect. The vertical line, which corresponds to the value 0 in the plot, is the line of no effect. Note that it says favours TICMT to the right of the vertical line and favours control therapy to the right of the vertical line.

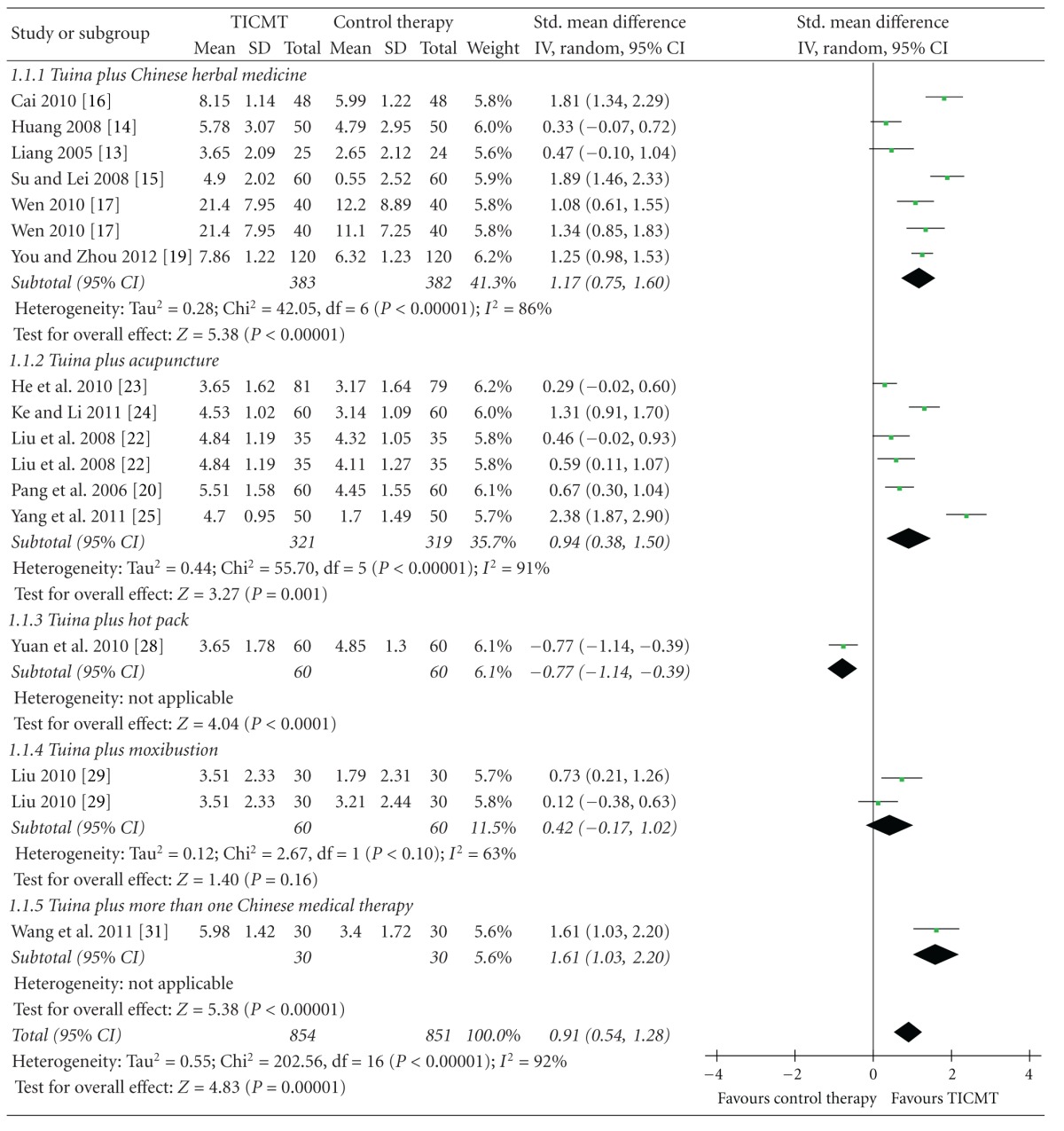

**Table 4:** Forest plots of the effect of Tuina-focused integrative Chinese medical therapies (TICMT) on functional status of in-patients with low back pain. Box in the line for each study: the mid-point of the box represents the mean effect estimate, which area shows the weight given to the study, and the line represents the confidence intervals of the mean effect estimate. The diamond below these studies represents the overall effect. The vertical line, which corresponds to the value 0 in the plot, is the line of no effect. Note that it says favours TICMT to the right of the vertical line and favours control therapy to the right of the vertical line.

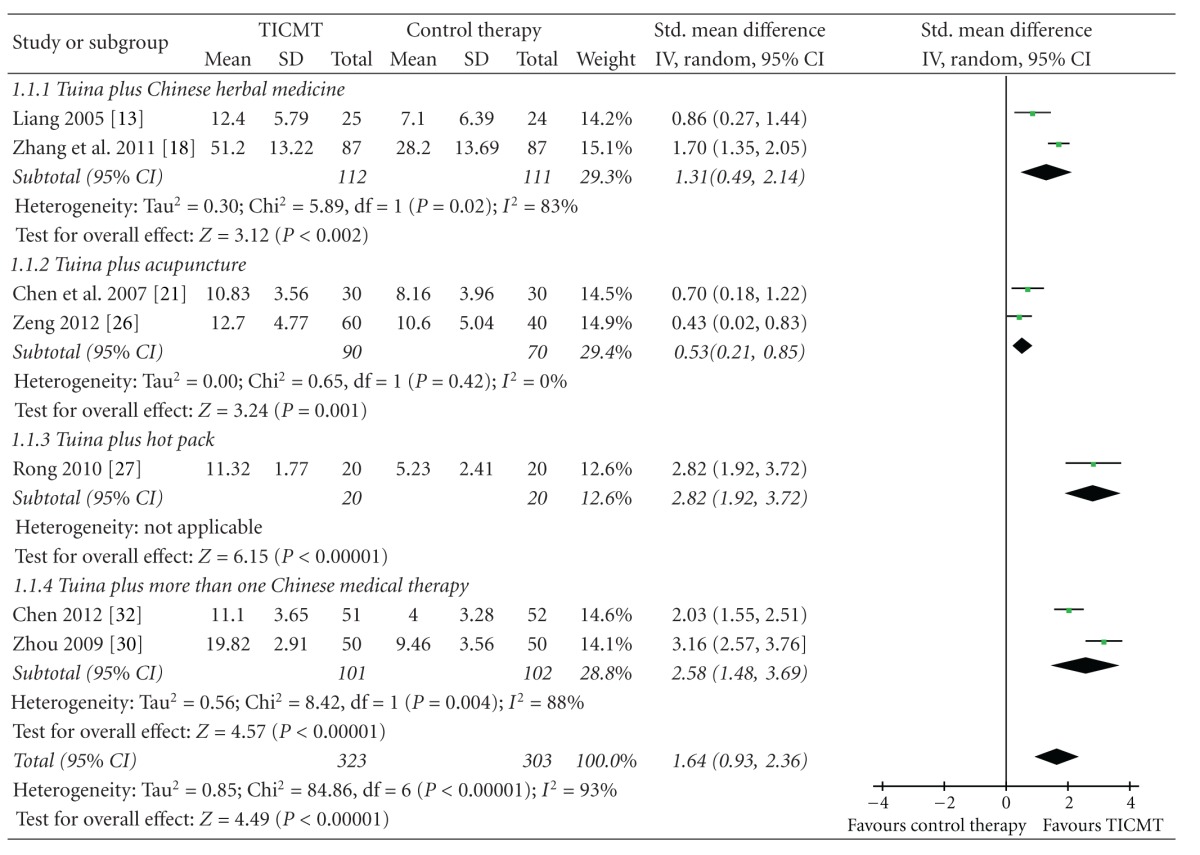

## Appendices

### A. Search Strategies

#### A.1. PubMed Publication Date from 2001/01/01 to 2012/06/30, Randomized Controlled Trial

1. ((((((low back pain [Title/Abstract]) OR back pain [Title/Abstract]) OR backache [Title/Abstract]) OR dorsalgia [Title/Abstract]) OR lumbago [Title/Abstract]) OR lumbar disc herniation [Title/Abstract]) OR lumbar sprain [Title/Abstract].
2. (((Tuina [Title/Abstract]) OR massage [Title/Abstract]) OR mobilization [Title/Abstract]) OR spinal manipulation [Title/Abstract].
3. ((((acupuncture [Title/Abstract]) OR electroacupuncture [Title/Abstract]) OR herbal medicine [Title/Abstract]) OR moxibustion [Title/Abstract]) OR hot pack [Title/Abstract].
4. ((1) AND (2)) AND (3).

#### A.2. EMBASE Publication Date from 2001/01/01 to 2012/06/30

1. (low back pain OR backache OR back pain OR dorsalgia OR lumbago OR lumbar disc herniation OR lumbar sprain).ti.
2. (Tuina OR massage OR mobilization OR spinal manipulation).ti.
3. (acupuncture OR electroacpuncture OR herbal medicine OR moxibustion OR hot pack).ti.
4. (random OR randomly OR randomized controlled trial OR RCT).ab.
5. (((1) AND (2)) AND (3)) AND (4).
6. (low back pain OR backache OR back pain OR dorsalgia OR lumbago OR lumbar disc herniation OR lumbar sprain).ab.
7. (Tuina OR massage OR mobilization OR spinal manipulation).ab.
8. (acupuncture OR electroacpuncture OR herbal medicine OR moxibustion OR hot pack).ab.
9. (((6) AND (7)) AND (8)) AND (4).

#### A.3. Cochrane Library Publication Date from 2001/01/01 to 2012/06/30

1. (“low back pain” OR “back pain” OR “backache” OR “dorsalgia” OR “lumbago” OR “lumbar disc herniation” OR “lumbar sprain” in title/abstract/keywords) AND (“Tuina” OR “massage” OR “mobilization” OR “spinal manipulation” in title/abstract/keywords) AND (“acupuncture” OR “electroacupuncture” OR “herbal medicine” OR “moxibustion” OR “hot pack” in title/abstract/keywords).

#### A.4. Chinese Databases including China Knowledge Resource Integrated Database (CNKI) Publication Date from 2001/01/01 to 2012/06/30

1. (low back pain [Title] OR back pain [Title] OR lumbar disc herniation [Title] OR lumbar sprain [Title]).
2. (Tuina [Title] OR massage [Title] OR mobilization [Title] OR spinal manipulation [Title]) AND (1).
3. (acupuncture [Title] OR electroacupuncture [Title] OR herbal medicine [Title] OR moxibustion [Title] OR hot pack [Title]) AND (2).
4. random [Abstract] AND (3).
5. (low back pain [Abstract] OR back pain [Abstract] OR lumbar disc herniation [Abstract] OR lumbar sprain [Abstract]).
6. (Tuina [Abstract] OR massage [Abstract] OR mobilization [Abstract] OR spinal manipulation [Abstract]) AND (5).
7. (acupuncture [Abstract] OR electroacupuncture [Abstract] OR herbal medicine [Abstract] OR moxibustion [Abstract] OR hot pack [Abstract]) AND (6).
8. random [Abstract] AND (7).

#### A.5. Weipu Database for Chinese Technical Periodicals (VIP) Publication Date from 2001/01/01 to 2012/06/30

1. (((low back pain [Title] OR back pain [Title] OR lumbar disc herniation [Title] OR lumbar sprain [Title]) AND (Tuina [Title] OR massage [Title] OR mobilizationi [Title] OR spinal manipulation [Title])) AND (acupuncture [Title] OR electroacupuncture [Title] OR herbal medicine [Title] OR moxibustion [Title] OR hot pack [Title])) AND random [Abstract].
2. (((low back pain [Abstract] OR back pain [Abstract] OR lumbar disc herniation [Abstract] OR lumbar sprain [Abstract]) AND (Tuina [Abstract] OR massage [Abstract] OR mobilizationi [Abstract] OR spinal manipulation [Abstract])) AND (acupuncture [Abstract] OR electroacupuncture [Abstract] OR herbal medicine [Abstract] OR moxibustion [Abstract] OR hot pack [Abstract])) AND random [Abstract].

#### A.6. Wanfang Data Publication Date from 2001/01/01 to 2012/06/30

1. (Title = low back pain OR back pain OR lumbar disc herniation OR lumbar sprain) AND (Title = Tuina OR massage OR mobilization OR spinal manipulation) AND (Title = acupuncture OR electroacupuncture OR herbal medicine OR moxibustion OR hot pack) AND (Abstract = random).
2. (Abstract = low back pain OR back pain OR lumbar disc herniation OR lumbar sprain) AND (Abstract = Tuina OR massage OR mobilization OR spinal manipulation) AND (Abstract = acupuncture OR electroacupuncture OR herbal medicine OR moxibustion OR hot pack) AND (Abstract = random).
